# Transcriptomic and Weighted Gene Co-expression Correlation Network Analysis Reveal Resveratrol Biosynthesis Mechanisms Caused by Bud Sport in Grape Berry

**DOI:** 10.3389/fpls.2021.690095

**Published:** 2021-06-18

**Authors:** Feng Leng, Yunling Ye, Jialing Zhou, Huijuan Jia, Xiaoheng Zhu, Jiayu Shi, Ziyue Zhang, Nan Shen, Li Wang

**Affiliations:** ^1^College of Horticulture and Plant Protection, Yangzhou University, Yangzhou, China; ^2^Key Laboratory of Horticultural Plant Growth, Development and Quality Improvement of the Ministry of Agriculture/Department of Horticulture, Zhejiang University, Hangzhou, China

**Keywords:** grape, resveratrol, transcriptome, ultra performance liquid chromatography-high resolution tandem mass spectrometry, weighted gene co-expression network analysis

## Abstract

Resveratrol is a natural polyphenol compound produced in response to biotic and abiotic stresses in grape berries. However, changes in resveratrol caused by bud sport in grapes are scarcely reported. In this study, *trans*-resveratrol and *cis*-resveratrol were identified and quantified in the grape berries of ‘Summer Black’ and its bud sport ‘Nantaihutezao’ from the veraison to ripening stages using ultra performance liquid chromatography-high resolution tandem mass spectrometry (UPLC-HRMS). We found that bud sport accumulates the *trans*-resveratrol earlier and increases the contents of *cis-resveratrol* in the earlier stages but decreases its contents in the later stages. Simultaneously, we used RNA-Seq to identify 51 transcripts involved in the stilbene pathways. In particular, we further identified 124 and 19 transcripts that negatively correlated with the contents of *trans*-resveratrol and *cis*-resveratrol, respectively, and four transcripts encoding F3'5'H that positively correlated with the contents of *trans*-resveratrol by weighted gene co-expression network analysis (WGCNA). These transcripts may play important roles in relation to the synergistic regulation of metabolisms of resveratrol. The results of this study can provide a theoretical basis for the genetic improvement of grapes.

## Introduction

**Figure 1:**
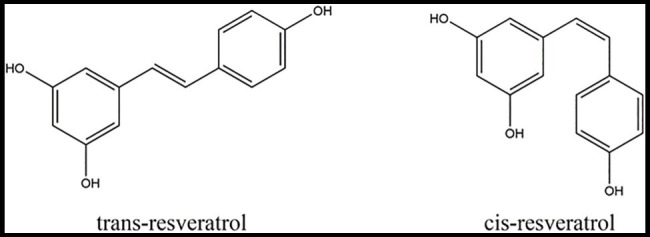
Chemical structures of *trans*-resveratrol and *cis*-resveratrol.

Resveratrol (C_14_H_12_O_3_; 3,4',5-trihydroxystilbene) is a beneficial secondary metabolite that belongs to the stilbene family, consisting of two aromatic rings joined by a methylene bridge, and exists in two isomers, *trans*-resveratrol and *cis*-resveratrol (Stervbo et al., 2007; Guerrero et al., 2010; Figure 1). The difference between the chemical structures of the *cis*-resveratrol and *trans*-resveratrol is the geometry of carbon–carbon double bond (Huang and Mazza, 2011). Resveratrol, in general, and *trans*-resveratrol, in particular, have many biological activities, including antifungal and antibacterial effects, as well as neuroprotective, cardioprotective, type 2 diabetes, and anticancer actions (Vitaglione et al., 2005; Yu et al., 2012; Sun et al., 2021). Apart from the above effects, resveratrol also has positive effects on the resistance of plants to biotic stress and can enhance the nutritional value of several vegetables and fruits, such as peanuts and grapes (Harikumar and Aggarwal, 2008; Giovinazzo et al., 2012).

Resveratrol is derived from the phenylpropanoid pathway that involves a series of enzymes such as phenylalanine ammonia-lyase (PAL), 4-coumarate: coenzyme A ligase (4CL), cinnamic acid 4-hydroxylase (C4H), and stilbene synthase (STS; Gatto et al., 2008; Wang et al., 2010; Duan et al., 2015). In grapes, *trans*-resveratrol is a leading and important stilbene compound, but *cis*-resveratrol has not been detected often (Liu et al., 2010). Many studies on resveratrol production have shown that its biosynthesis is induced by biotic and abiotic stresses, for example, UV-C radiation (Bais et al., 2000; Bai et al., 2019), pathogen attack (Schnee et al., 2008), salinity stress (Ismail et al., 2012), and application of chemicals, such as aluminum ions and ozone (Adrian et al., 1996; Duan et al., 2015; Bai et al., 2019). It can be induced in response to plant hormones, such as ethylene and jasmonates (Belhadj et al., 2008; D’Onofrio et al., 2009). Moreover, resveratrol can be transported to different tissues of grapevine subjected to stress and adaptation mechanisms in response to the environment, which may cause resveratrol to be converted to its isomers (Ji et al., 2014). Transcriptome analysis provides an insight into the *PAL*, *4CL*, and *STS* expression profiles showing that the concentration of their transcripts increased from veraison to maturity, as well as the content of resveratrol also increases in the grape (Gatto et al., 2008). Meanwhile, the competition between STS and chalcone synthase (CHS) for the same substrates is the factor that affects the accumulation of resveratrol (Jeandet et al., 1995; Gatto et al., 2008; Yin et al., 2016; Lu et al., 2021).

Bud sport is a consequence of the genetic variation of somatic cells leading to the occurrence of qualitative and quantitative phenotypic alterations in many vegetatively propagating plants, including grapes (Zhao et al., 2015). It is an essential source for breeding new varieties when bud sports grow into branches in fruit trees. Among bud sports, most studies have focused on the molecular basis of the color changes and fruit ripening of grape (Guo et al., 2016; Bustamante et al., 2017; Xu et al., 2017). Bustamante et al. (2017) found a novel earlier ripening and sweeter taste bud mutation ‘Red Globe’, registered as ‘Pink Globe’. Compared to its parents, ‘Pink Globe’ has lower anthocyanin content and *Vvufgt* expression level. A similar result exists between ‘Benitaka’ and its bud mutation ‘Brazil’ (Xu et al., 2017). Guo et al. (2016) revealed many genes that were differentially expressed in the berries of ‘Kyoho’ and its early ripening mutant ‘Fengzao’ by using comparative profiling analysis. However, a comprehensive analysis of resveratrol being affected by bud sport is still lacking to date. Recently, we have identified a novel early ripening bud mutation on ‘Summer Black’ and named it ‘Nantaihutezao’ in Huzhou, Zhejiang Province, the maturity time of which is approximately 2 weeks earlier than ‘Summer Black’. There were no significant differences in the total anthocyanin contents and total soluble solid (TSS) between the two cultivars (Leng et al., 2021). We previously found that the resveratrol content and its composition in berry peel were different between the two cultivars, which might constitute a useful experimental system for the study of resveratrol metabolic mechanism and will also help us to better understand the mechanism of grape ripening underlying the bud sport variety. In this study, we performed resveratrol quantification and transcriptomic comparison between two cultivars at six time points, from veraison to ripening stages, to investigate the genetic difference. We aimed to find candidate genes related to the biosynthesis of *trans*-resveratrol and *cis*-resveratrol, which will lay a foundation for further identification of the function of genes and their improvement for fruit quality.

## Materials and Methods

### Sample Collection

A 3-year-old ‘Summer Black’ and its bud sport ‘Nantaihutezao’ grape berries were used as the materials in this study. The grapevines were planted in a plastic film greenhouse in Longcong village of Huzhou city in the Zhejiang province, with normal fertilizer and water management along with the control of pests and diseases. Samples were collected every 7 days from veraison (S5) to berry ripening (S10). Thirty grape berries were picked from two clusters in different positions and directions each time with no evidence of stress symptoms or disease. At the same time, the samples were immediately frozen in liquid nitrogen and then stored at −80°C for subsequent testing.

### Resveratrol Profiling

Resveratrol was extracted according to a previously published method. The extracted resveratrol solution was analyzed and quantified by UPLC-HRMS and ultra-high-performance liquid chromatography-diode array detector (UPLC-DAD), respectively, following the established methods in our laboratory (Leng et al., 2020).

### RNA Extraction and RNA-Seq

Total RNAs of pericarp were extracted using Trizol (Invitrogen) with three biological replicates according to the recommendations of the manufacturer. The RNA quantity was checked using the NanoDrop ND-1000 spectrophotometer (NanoDrop Technologies, US), the RNA concentration was assessed using Qubit® RNA Assay Kit in Qubit®2.0 Fluorometer (Life Technologies, US), and the RNA integrity was evaluated using an Agilent 2,100 Bioanalyzer (Agilent Technologies, US). A total of 36 cDNA libraries (three biological repetitions) were constructed using TruSeq™ RNA Sample Preparation Kit (Illumina, US) following the instructions of the manufacturer and deep sequenced by the Biomarker Biotechnology Corporation (Beijing, China), using Illumina HiSeq X-ten platform. All the raw sequence data have been deposited at NCBI Sequence Read Archive (SRA) with the accession code GSE142313.

### Gene Functional Annotation and Expression Analysis

After removing adapter reads and filtering low-quality sequences, the clean reads were aligned to the reference *Vitis vinifera* genome (Jaillon et al., 2007) using the TopHat 2 software(Trapnell et al., 2009).^1^ The fragments per kilobase of exon per million mapped reads (FPKM) were used to calculate the abundance values of the transcript using the Cuffdiff program.^2^ Differentially expressed genes between the two cultivars at the same stage were analyzed using DESeq software (Anders and Huber, 2010), with the estimated absolute log_2_ fold change (FC) > 1 and false discovery rate (FDR) < 0.05. Kyoto Encyclopedia of Genes and Genomes (KEGG) pathway enrichment analysis was performed using KOBAS 3.0 software (Wu et al., 2006; Xie et al., 2011).

### Weighted Gene Co-expression Network Analysis and Visualization

From all genes, the interesting gene modules and co-expression network analysis were identified by weighted gene co-expression network analysis (WGCNA) package in R (Langfelder and Horvath, 2008). The sample trait based on the correlation of the gene expression profile with intramodular connectivity and gene significance were used to identify critical genes in two cultivars at the different phases for further validation. A soft thresholding power of seven in the WGCNA package was prescribed as a soft threshold of the correlation matrix. Topological overlap matrix (TOM) similarity algorithm was used to convert the adjacency matrix to TOM and to hierarchically cluster genes. The Dynamic Hybrid Tree Cut algorithm was adopted to cut the hierarchal clustering tree and define modules as branches from the tree cutting (Zhan et al., 2015). Module Eigen gene was used to summarize the expression profile of each module.

### Real-Time Quantitative Reverse Transcription -PCR Validation and Statistical Analysis

Real-time quantitative reverse transcription (qRT)-PCR analysis was performed to validate the RNA-Seq data according to a previous method (Leng et al., 2017). The primers are listed in Supplementary Table S1. *Glyceraldehyde 3-phosphate dehydrogenase* (GAPDH: NCBI accession number: CBI14856.3) gene was used as the internal control for calculating the relative expression of the mRNA. All samples were analyzed in triplicate, and the results were presented as the mean ± SE. The statistical significance of differences was determined with a t-test using SPSS16.0 statistical software package (IBM). Originpro 2016 (Microcal Software) was applied for the construction of the figures.

## Results

**Figure 2:**
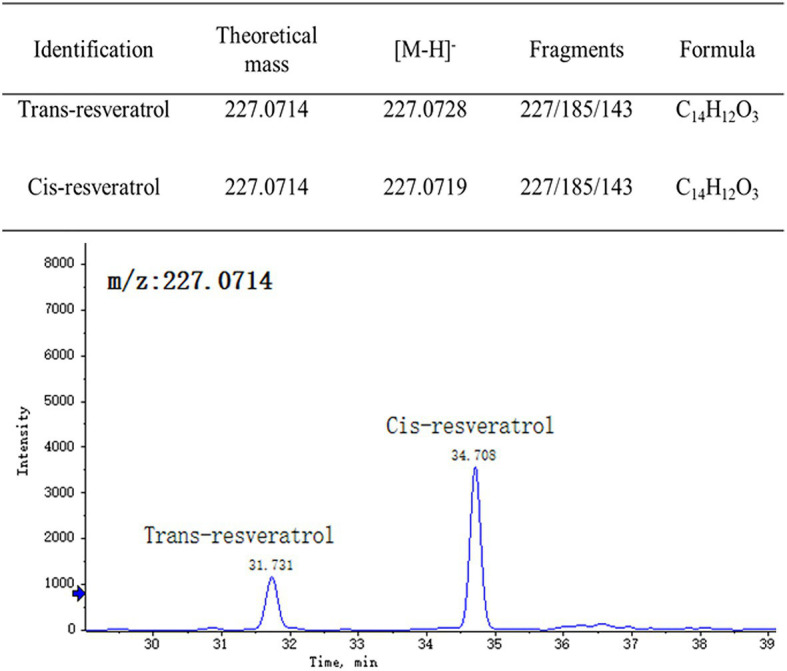
Chromatographic and mass spectrometric parameters of detected *trans*-resveratrol and *cis*-resveratrol in grape berry.

*Trans*-resveratrol and *cis*-resveratrol in our samples were identified by using reversed-phase UPLC-HRMS, the ESI-ion trap MS and MS/MS data were compared with those found in the previous studies, and the retention times were compared with that of the standards (Figure 2). The tandem mass spectrum of the [M−H]^−^ ion at *m/z* 227 for both *trans*-resveratrol and *cis*-resveratrol, and their fragmentation spectrum were dominated by the product ion at *m/z* 185 and 143, representing the loss of one and two ketene molecule. The retention times of *trans*-resveratrol and *cis*-resveratrol under the established conditions were 31.73 and 34.708 min, respectively.

**Figure 3:**
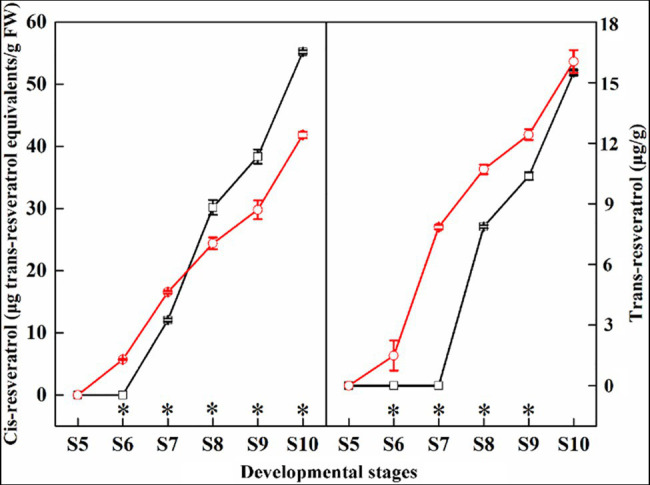
The changes in the contents of *trans*-resveratrol and *cis*-resveratrol during grape berry development and ripening in ‘Summer Black’ (black line) and ‘Nantaihutezao’ (red line). ^*^Indicates the significant differences (*p* < 0.05, *n* = 3).

In this study, we compared the contents of *trans*-resveratrol and *cis*-resveratrol in the ‘Summer Black’ and ‘Nantaihutezao’ grape berry skins from veraison to ripening stages (Figure 3). The results showed that both the contents of *trans*-resveratrol and *cis*-resveratrol were accumulated with increasing time. Compared with the ‘Summer Black’, the contents of *cis*-resveratrol were higher in ‘Nantaihutezao’ at the S6 and S7 stages, but lower in the later stages. For *trans*-resveratrol, the contents in ‘Nantaihutezao’ were higher than ‘Summer Black’ during the development, but no difference in maturity. In other words, bud sport only leads to earlier accumulation of *trans*-resveratrol without increasing its contents.

**Figure 4:**
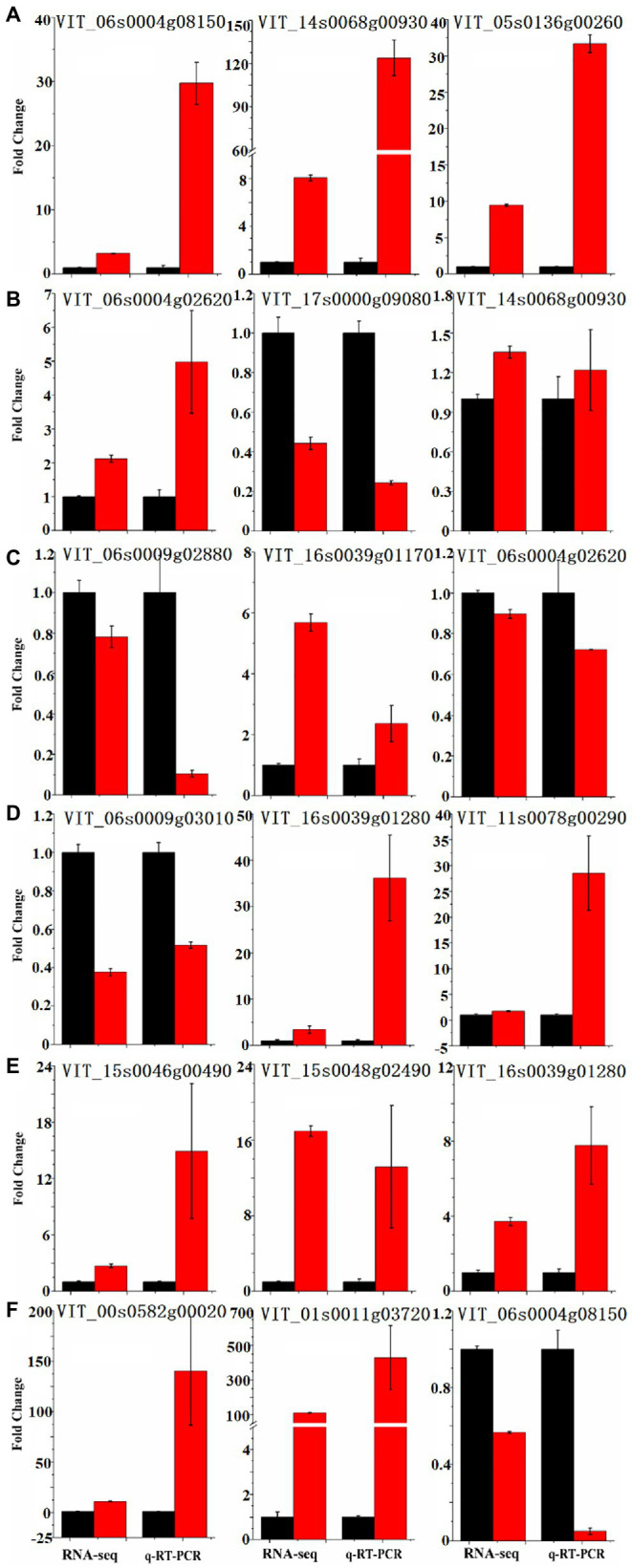
Real-time quantitative reverse transcription (qRT)-PCR validation of the differentially expressed genes between ‘Summer Black’ (black bar chart) and its bud sport ‘Nantaihutezao’ (red bar chart). **(A)** S5; **(B)** S6; **(C)** S7; **(D)** S8; **(E)** S9; and **(F)** S10.

As the metabolism of resveratrol of the bud sport changed in this study, we performed a transcriptomic comparison between ‘Summer Black’ and ‘Nantaihutezao’ cultivars from veraison to ripening stages of grape berry to gain insights into the molecular mechanism related to the metabolism of resveratrol caused by bud sport and identify key regulatory genes. RNA-Seq was performed using the Illumina HiSeq X-ten sequencing platform. A total of 273.05 Gb clean data were obtained following quality assessment and data filtering, with more than 90% Q30 scores of clean bases, and the mapped ratios were between 66.21 and 73.65% (Leng et al., 2021). To confirm the availability of data obtained by RNA-Seq in this study, some upregulated and downregulated genes between two cultivars were selected for quantification by qRT-PCR. Those genes encoded members of the enzymes involved in the phenylpropanoid pathway, transcription factors, and some genes randomly selected for different expression levels in each stage (Figure 4). The results indicated that the qRT-PCR expression profiles were generally consistent with the RNA-Seq values, suggesting the reliability of the RNA-Seq data.

**Figure 5:**
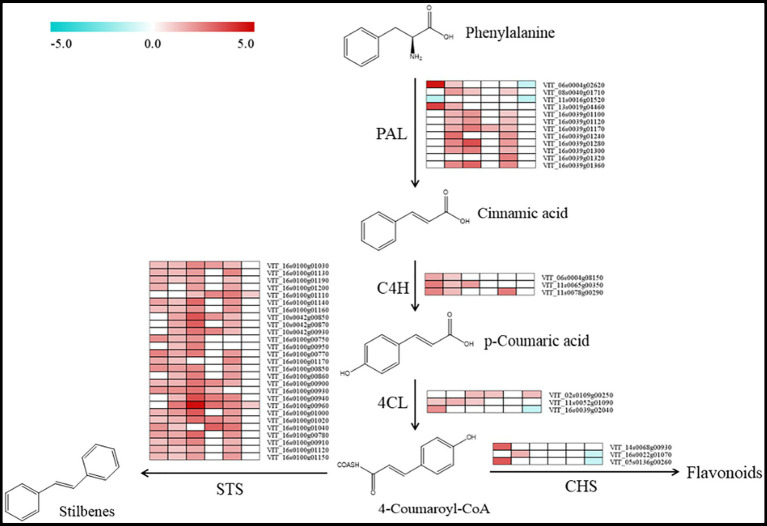
Biosynthetic pathway of resveratrol in grape berries. Boxes from left to right that follow the berry development were obtained by using the MultiExperiment Viewer software. The data set comprised of data regarding the changes in the relative expression of the ‘Nantaihutezao’ in relation to the ‘Summer Black’ at the same stage, which were expressed as log_2_ fold change.

According to the KEGG annotations, we found 45 differentially expressed transcripts involved in the biosynthesis of stilbene induced by bud sport during grape berry development (Figure 5). Among them, there were 27 transcripts with 26 upregulated genes and 1 downregulated gene, 39 transcripts with all upregulated genes, 35 transcripts with all upregulated genes, 12 transcripts with all upregulated genes, 34 transcripts with all upregulated genes, and 6 transcripts with 3 upregulated genes and 3 downregulated genes in the phases of S5, S6, S7, S8, S9, and S10, respectively. These genes may play key roles in the variations of *trans*-resveratrol and *cis*-resveratrol contents. In addition, three transcripts (*VIT_14s0068g00930*, *VIT_16s0022g01070*, and *VIT_05s0136g00260*) encoding CHS may play an important role in the synergistic regulation of metabolisms of resveratrol, which needs to be further analyzed in the future.

**Figure 6:**
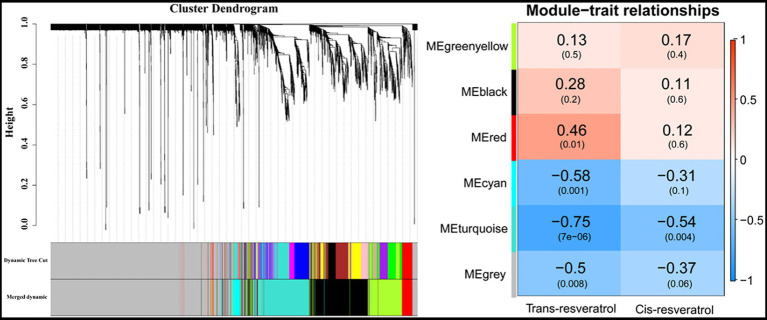
The relationship between the module and the contents of *trans*-resveratrol and *cis*-resveratrol was performed by weighted gene co-expression network analysis (WGCNA).

To get a broader view on the gene expression levels involved in *trans*-resveratrol and *cis*-resveratrol metabolism pathways, respectively, modules associated with the contents of *trans*-resveratrol and *cis*-resveratrol were obtained by WGCNA (Figure 6). In this study, six main modules were identified from the RNA-Seq data. The red module contained 150 genes that were positively correlated with the contents of *trans*-resveratrol (cor = 0.46, *p* = 0.01). The modules that negatively correlated with the contents of *trans*-resveratrol were cyan (cor = −0.58, *p* = 0.001), turquoise (cor = −0.75, *p* = 7e-06), and gray (cor = −0.5, *p* = 0.008). The numbers of genes in each module are 87, 647, and 2,109 for cyan, turquoise, and gray colors, respectively. Interestingly, the turquoise module was also negatively correlated with the contents of *cis*-resveratrol (cor = −0.54, *p* = 0.004).

**Figure 7:**
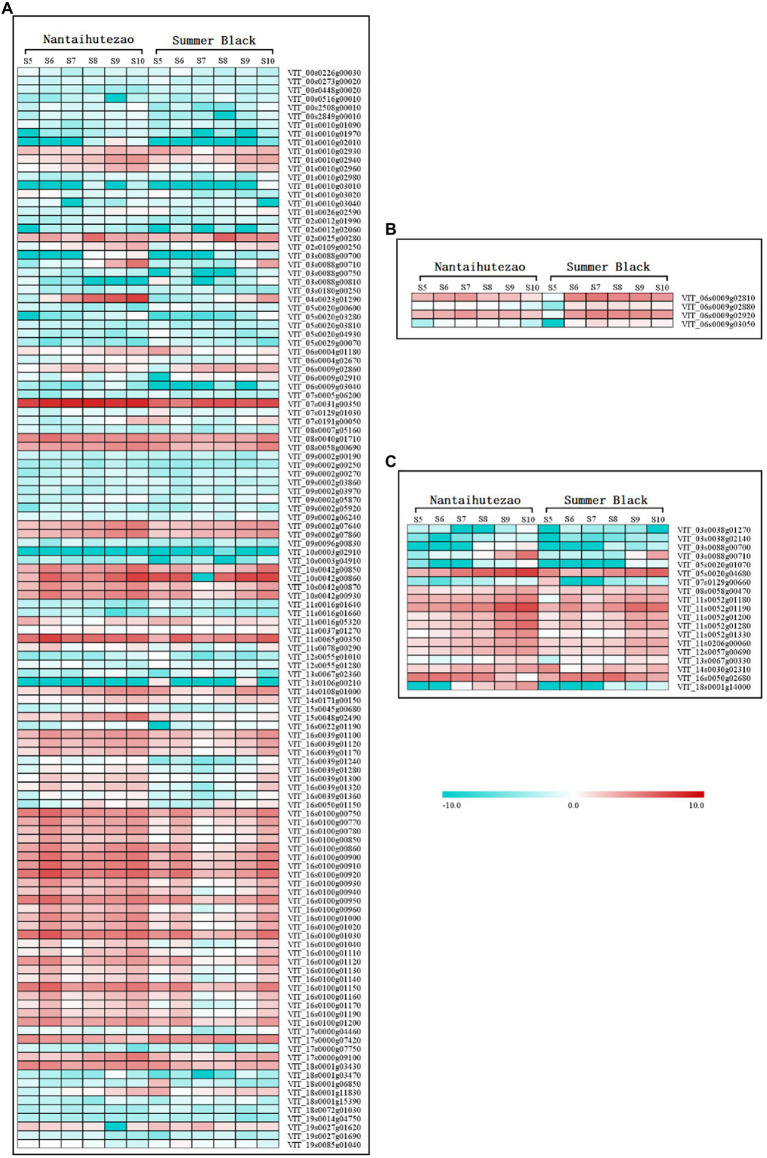
The expression profiles of genes enriched in the different Kyoto Encyclopedia of Genes and Genomes (KEGG) pathways in the modules correlated with the contents of resveratrol. Boxes from left to right that follow the berry development were obtained by using the MultiExperiment Viewer software. The data set was normalized to the values as log_2_ transformed. The left set of boxes is for the bud sport ‘Nantaihutezao’, and the right set is for ‘Summer Black’. **(A)** Genes negatively correlated with the contents of *trans*-resveratrol; **(B)** genes positively correlated with the contents of *trans*-resveratrol; and **(C)** genes negatively correlated with the contents of *cis*-resveratrol.

The genes in the red, cyan, turquoise, and gray modules were found to be significantly correlated with the contents of *trans*-resveratrol and *cis*-resveratrol. The association of separate genes within these modules with the contents of *trans*-resveratrol and *cis*-resveratrol was further investigated. For this purpose, we performed individual pathway enrichment analyses for these genes in the significantly correlated modules to hunt their related functions and obtain the significant KEGG pathways (Supplementary Figure S1). In this study, our analysis revealed 73 candidate transcripts enriched in the phenylpropanoid pathway that negatively correlated with the contents of *trans*-resveratrol. In addition, there are 51 transcripts enriched in the flavonoid biosynthesis and plant–pathogen interaction pathways, which may play a negative regulatory role in the metabolism of resveratrol (Figure 7A; Supplementary File S1). Furthermore, we found four transcripts *VIT_06s0009g02810*, *VIT_06s0009g02880*, *VIT_06s0009g02920*, and *VIT_06s0009g03050* encoding flavonoid-3'5'-hydroxylase (F3'5'H) to be positively correlated with the contents of *trans*-resveratrol (Figure 7B; Supplementary File S2). In this study, we have found no genes that positively correlated with the contents of *cis*-resveratrol, but 19 candidate genes enriched in the plant hormone signal transduction pathway negatively correlated with the contents of *cis*-resveratrol, suggesting that these genes may participate in the biosynthesis of *cis*-resveratrol, but further study needs to be undertaken to determine their potential roles (Figure 7C; Supplementary File S3). Generally, these findings have revealed promising candidate genes for contributing to the variation in the contents of *trans*-resveratrol and *cis*-resveratrol.

## Discussion

Many studies previously reported that the accumulation of resveratrol is well known in grape berries at veraison and continues throughout berry ripening, with significant differences among grape varieties (Gatto et al., 2008; Mei et al., 2015). In the cultivation of grapes, we discovered a bud sport on ‘Summer Black’ and named it ‘Nantaihutezao’, whose maturity time is 2 weeks earlier than that of its parents. Besides, during preliminary observation, ‘Nantaihutezao’ showed better color and fruit quality than ‘Summer Black’ (Leng et al., 2021). In terms of resveratrol, the contents of *trans*-resveratrol and *cis*-resveratrol were both accumulated with increasing time, which is consistent with previous findings. Interestingly, the contents and rates of *trans*-resveratrol and *cis*-resveratrol accumulation were significantly different due to bud sport. Considering these changes, we performed transcriptomic comparison and WGCNA to gain insights into the molecular mechanism related to the metabolism of resveratrol caused by bud sport and identify key regulatory genes.

The phenylalanine is known as the primary substrate for the biosynthesis of flavonoids and stilbenes *via* the phenylpropanoid pathway in most plants such as grapes (Jeandet et al., 2021; Lu et al., 2021). The biosynthesis of resveratrol was achieved through a small branch of the phenylpropanoid pathway and is considered as a competitive extension of the branch of the flavonoids (Vannozzi et al., 2018; Andi et al., 2021; Jeandet et al., 2021). Upstream of the phenylpropanoid pathway, the expression levels of *PAL*, *C4H*, *4CL*, and *STS* genes were closely related to the contents of resveratrol. In this study, we found 12 transcripts (*VIT_06s0004g02620*, *VIT_08s0040g01710*, *VIT_11s0016g01520*, *VIT_13s0019g04460*, *VIT_16s0039g01100*, *VIT_16s0039g01120*, *VIT_16s0039g01170*, *VIT_16s0039g01240*, *VIT_16s0039g01280*, *VIT_16s0039g01300*, *VIT_16s0039g01320*, and *VIT_16s0039g01360*) encoding PAL, 3 transcripts (*VIT_06s0004g08150*, *VIT_11s0065g00350*, and *VIT_11s0078g00290*) encodeing C4H, and 3 transcripts (*VIT_02s0109g00250*, *VIT_11s0052g01090* and *VIT_16s0039g02040*) encoding 4CL that were significantly upregulated or downregulated at some developmental stages between two cultivars. These differentially expressed transcripts might be involved in regulating the biosynthesis of resveratrol. STS is a key enzyme leading to the biosynthesis of resveratrol (Flamini et al., 2013; Lavhale et al., 2018; Vannozzi et al., 2018). We found 27 transcripts (*VIT_16s0100g01030*, *VIT_16s0100g01130*, *VIT_16s0100g01190*, *VIT_16s0100g01200*, *VIT_16s0100g01110*, *VIT_16s0100g01140*, *VIT_16s0100g01160*, *VIT_10s0042g00850*, *VIT_10s0042g00870*, *VIT_10s0042g00930*, *VIT_16s0100g00750*, *VIT_16s0100g00950*, *VIT_16s0100g00770*, *VIT_16s0100g01170*, *VIT_16s0100g00850*, *VIT_16s0100g00860*, *VIT_16s0100g00900*, *VIT_16s0100g00930*, *VIT_16s0100g00940*, *VIT_16s0100g00960*, *VIT_16s0100g01000*, *VIT_16s0100g01020*, *VIT_16s0100g01040*, *VIT_16s0100g00780*, *VIT_16s0100g00910*, *VIT_16s0100g01120*, and *VIT_16s0100g01150*) encoding STS that were differentially expressed at certain developmental stages, and all of them were upregulated in Nantaihutezao. These transcripts might be involved in the metabolism of resveratrol. Furthermore, CHS, a key enzyme of the flavonoid pathway, is closely related to STS and also affects the resveratrol content, because both enzymes compete for the same substrate and control the entry points into the flavonoid and stilbene pathways, respectively (Andi et al., 2021; Jeandet et al., 2021). In our data, it was found that three differentially expressed transcripts (*VIT_14s0068g00930*, *VIT_16s0022g01070*, and VIT_05s0136g00260) encoding CHS might be related to the changes in the resveratrol. For the *trans*-resveratrol, many previous studies have reported a relationship in the high contents with increasing concentration of some genes, such as *PAL*, *4CL*, and *STS*, in the grape varieties from veraison to maturity (Degu et al., 2014; Duan et al., 2015; Suzuki et al., 2015; Wong et al., 2016; Bai et al., 2019). Conversely, *cis*-resveratrol was neglected because it has not been detected often, but it appears to be formed by hydrolysis of pieced or resveratrol polymers. It can also be transformed from *trans*-resveratrol by light-induced or enzymatic isomerization and may possess health-promoting properties (Lopez-Hernandez et al., 2007; Liu et al., 2011; Erte et al., 2020). Early studies indicated that the contents of *trans*-resveratrol and *cis*-resveratrol increase from veraison to harvesting and are induced in response to biotic and abiotic stresses. Meanwhile, *PAL*, *4CL*, and *STS* expression profiles showed an increasing concentration of their transcripts and had a higher accumulation in the grape of high resveratrol contents (Gatto et al., 2008; Bai et al., 2019). Anyway, our transcriptome analysis showed that 48 differentially expressed transcripts, encoding *PAL*, *C4H*, *4CL*, *STS*, and *CHS*, were important genes in the biosynthesis of stilbene during grape berry development induced by bud sport (Figure 5). Therefore, we speculated that these transcripts might play important roles in the accumulation of resveratrol.

In recent years, the accumulation of resveratrol induced by biotic and abiotic stresses, such as pathogen attack and plant hormones, has been demonstrated by many studies (Belhadj et al., 2008; Schnee et al., 2008; D’Onofrio et al., 2009). *WRKY*, *MYB*, *bZIP*, and *ERF* transcription factors have been involved in regulating *STS* genes to increase the resveratrol content (Riccio et al., 2020; Wang et al., 2020). In addition, *PRXs* genes also have been reported to participate in regulating stilbene synthesis (Park et al., 2021). Meanwhile, the phenylpropanoid pathway starts from phenylalanine, which belongs to the downstream pathway after the sucrose synthesis and degradation, glycolysis, and tricarboxylic acid cycle pathways (Dai et al., 2013). Upstream genes in those pathways also affect the resveratrol content. To further study the molecular mechanism of the biosynthesis of *trans*-resveratrol and *cis*-resveratrol, we performed WGCNA analysis to investigate functional modules that correlate with the contents of *trans*-resveratrol and *cis*-resveratrol and then enrich the genes in these functional modules into KEGG pathways. Our analysis has revealed 124 candidate transcripts enriched in the phenylpropanoid, flavonoid biosynthesis, and plant–pathogen interaction pathways (Supplementary File S1), which were proposed to play a negative regulatory role in the contents of *trans*-resveratrol. Among these candidate transcripts, most of them are annotated into metabolism and biosynthesis of phenylalanine, such as *VIT_19s0014g04750*, *VIT_05s0020g03280*, *VIT_17s0000g09100*, *VIT_16s0100g01130*, *VIT_16s0100g01030*, and *VIT_00s2508g00010*. *VIT_19s0014g04750* is annotated as beta-glucosidase that is involved in the phenylpropanoid biosynthesis pathway. It might affect the contents of *trans*-resveratrol by regulating its precursor, phenylalanine. *VIT_05s0020g03280* and *VIT_17s0000g09100* encoded primary-amine oxidase, which performs a similar function. *VIT_16s0100g01130* and *VIT_16s0100g01030* encoded STS, and *VIT_00s2508g00010* encoded PAL, which are directly involved in the stilbene biosynthesis pathway. Besides, numerous other transcripts are annotated into plant hormone signal transduction and plant–pathogen interaction pathways, such as *VIT_12s0055g01280*, *VIT_03s0088g00810*, and *VIT_05s0020g04930*. These transcripts might indirectly affect the contents of *trans*-resveratrol due to changes in plant hormones or pathogen attack. We found four transcripts (*VIT_06s0009g02810*, *VIT_06s0009g02880*, *VIT_06s0009g02920*, and *VIT_06s0009g03050*) encoding F3′5′H that were positively correlated with the contents of *trans*-resveratrol (Supplementary File S2), which belong to the biosynthesis of flavonoid. Because the competition between these genes in the biosynthesis of flavonoid and resveratrol for the same substrates was the factor to affect the resveratrol accumulation, similar to *CHSs*, we speculated that these enriched transcripts may have the potential role in the direct or indirect regulation of biosynthesis of *trans*-resveratrol. In addition, there are 19 candidate transcripts (*VIT_03s0038g01270*, *VIT_03s0038g02140*, *VIT_03s0088g00700*, etc.) enriched in the plant hormone signal transduction pathway (Supplementary File S3) that may participate in the biosynthesis of *cis*-resveratrol.

In conclusion, our findings provide hundreds of differentially expressed transcripts with obscure functions, which can provide new insights into the response of grape berries to bud sport, and also could be used in future functional and molecular biological studies of resveratrol metabolism.

## Data Availability Statement

The datasets presented in this study can be found in online repositories. The names of the repository/repositories and accession number(s) can be found in the article/Supplementary Material.

## Author Contributions

FL and LW designed the experiments and composed the study. FL, YY, JZ, XZ, and NS performed the experiments. FL, JS, and ZZ analyzed the data. FL and HJ contributed to reagents, materials, and analytical tools. All authors contributed to the article and approved the submitted version.

### Conflict of Interest

The authors declare that the research was conducted in the absence of any commercial or financial relationships that could be construed as a potential conflict of interest.

## Footnotes

^1^http://tophat.cbcb.umd.edu/

^2^http://cufflinks.cbcb.umd.edu/

## Supplementary Material

Supplementary Figure S1 Kyoto Encyclopedia of Genes and Genomes (KEGG) pathway of gene enrichment analysis.

Supplementary Table S1 Primer sequences of qRT-PCR.

Supplementary File S1 KEGG pathway enrichment analyses of genes was negatively correlated with trans-resveratrol.

Supplementary File S2 KEGG pathway enrichment analyses of genes was positively correlated with trans-resveratrol.

Supplementary File S3 KEGG pathway enrichment analyses of genes was negatively correlated with cis-resveratrol.
