# Supplementary figures and images for: Transcriptomic and Weighted Gene Co-expression Correlation Network Analysis Reveal Resveratrol Biosynthesis Mechanisms Caused by Bud Sport in Grape Berry
Source: Front Plant Sci. 2021 Jun 18;12:690095. doi: 10.3389/fpls.2021.690095 (PMC8253253; doi:10.3389/fpls.2021.690095)

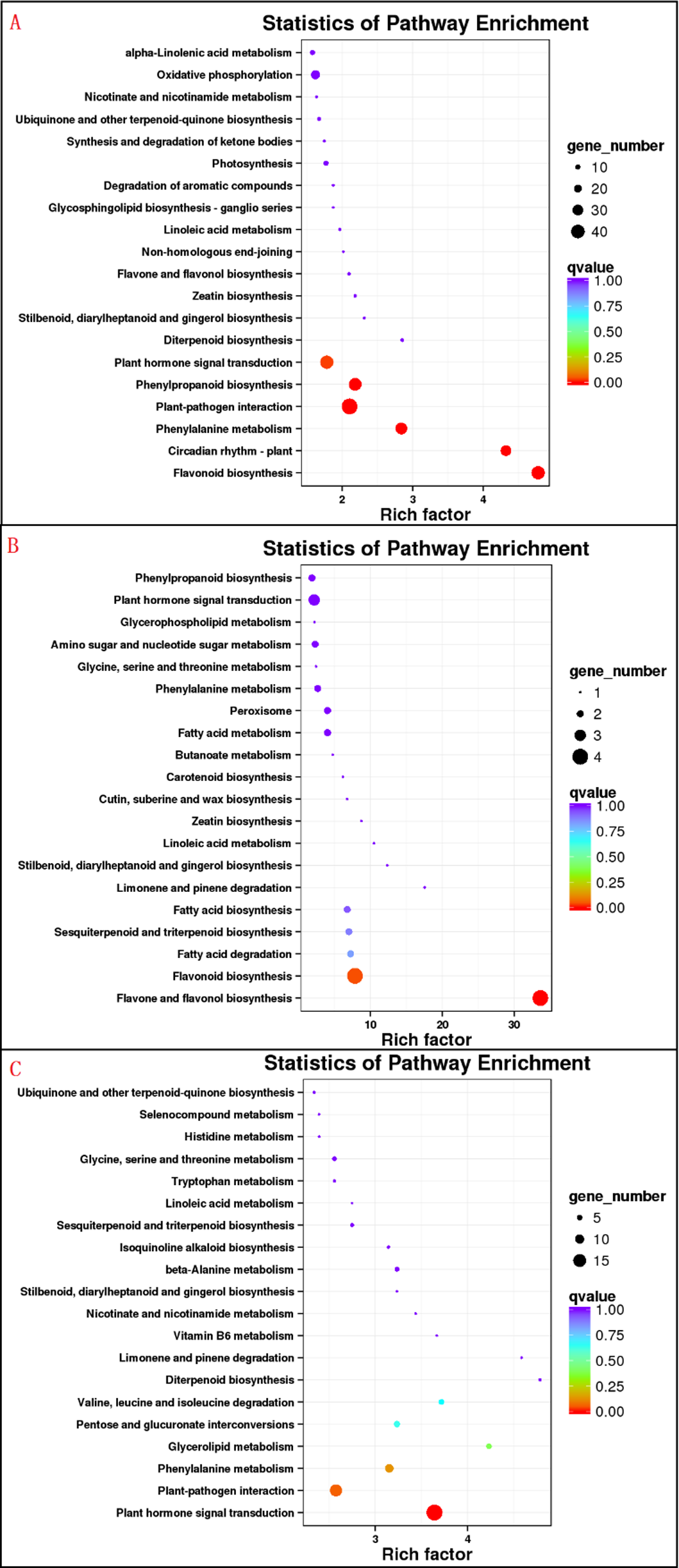

Supplement: Supplementary Figure S1 — Kyoto Encyclopedia of Genes and Genomes (KEGG) pathway of gene enrichment analysis. [file Image_1.TIF]
