# Supplementary material for: Transcriptomic and Weighted Gene Co-expression Correlation Network Analysis Reveal Resveratrol Biosynthesis Mechanisms Caused by Bud Sport in Grape Berry
Source: Front Plant Sci. 2021 Jun 18;12:690095. doi: 10.3389/fpls.2021.690095 (PMC8253253; doi:10.3389/fpls.2021.690095)
Supplement: Supplementary Table S1 — Primer sequences of qRT-PCR. [file Table_1.DOCX]

**Supplemental Table S1.** Primer sequences of qRT-PCR

| **Gene ID** | **Forward primer (5’–3’)** | **Reverse primer (3’–5’)** |
| --- | --- | --- |
| *VIT_06s0004g08150* | ACCACCTGAACCTCTCCGACTTAG | TTCTTGTGCGTGATCCGAACTCC |
| *VIT_14s0068g00930* | CGGCACGTTCTGAGCGAGTATG | ACCGTGAGACCTGGTCCGAATC |
| *VIT_05s0136g00260* | TGTAACAGCCACTCCTCCACCTC | GGCATTGCGAACTTCGTTGACG |
| *VIT_06s0004g02620* | TCTCACACCACAACGGCAACG | GTCCGCCACCATTCTCTTCACC |
| *VIT_17s0000g09080* | CTGCATCAGGTTCTAGGCAACAGG | CTGGCAATTGTGCTGCAATCTGAG |
| *VIT_06s0009g02880* | GAGTTCAAGGACATGGTGGTGGAG | GCCGTGTGCTCCTCCATCATC |
| *VIT_16s0039g01170* | CGTGAGTGATCCGCTGAACTGG | CACCACCGAGCCGAACAACC |
| *VIT_06s0009g03010* | GAGTTCAAGGACATGGTGGTGGAG | GCTGATGCCGTGTGCTCCTC |
| *VIT_16s0039g01280* | GAAGAGCTTCTGCGTGAGTGATCC | CACCACCGAGCCGAACAACC |
| *VIT_11s0078g00290* | CCAACCTCCACCAACTTCCATACC | ACCAGGCATTAACCACCACCTTG |
| *VIT_15s0046g00490* | GAGGCTCTCCCAACTTTGCC | TTCTCGACCCAAGCCCATCT |
| *VIT_15s0048g02490* | AGCCAATAATGGAGCCTTCAGCAG | AGTCACCAATTGCATGGCGTAGG |
| *VIT_16s0039g01280* | GAAGAGCTTCTGCGTGAGTGATCC | CACCACCGAGCCGAACAACC |
| *VIT_00s0582g00020* | ACAGCCGCACCTCCATCCTC | GGTGGTGGTGTCATCGTCAGAAC |
| *VIT_01s0011g03720* | TGGAACTGATGAACAACCGGAGTG | GACAGCACTTAGACCTCCAGCATG |
